# CRISPR–Cas systems and applications for crop bioengineering

**DOI:** 10.3389/fbioe.2024.1483857

**Published:** 2024-10-16

**Authors:** Mireia Uranga, Ana Montserrat Martín-Hernández, Nico De Storme, Fabio Pasin

**Affiliations:** ^1^ Laboratory for Plant Genetics and Crop Improvement, Division of Crop Biotechnics, Department of Biosystems, KU Leuven, Leuven, Belgium; ^2^ KU Leuven Plant Institute (LPI), KU Leuven, Leuven, Belgium; ^3^ Centre for Research in Agricultural Genomics (CRAG), Barcelona, Spain; ^4^ Institut de Recerca i Tecnologia Agroalimentàries (IRTA), Barcelona, Spain; ^5^ Instituto de Biología Molecular y Celular de Plantas (IBMCP), Consejo Superior de Investigaciones Científicas-Universitat Politècnica de València (CSIC-UPV), Valencia, Spain; ^6^ Centro de Investigaciones Biológicas Margarita Salas (CIB), Consejo Superior de Investigaciones Científicas, Madrid, Spain

**Keywords:** transgene-free genome editing, genome engineering, crop trait improvement, precise breeding, CRISPR (clustered regularly interspaced short palindromic repeat), Cas9 (CRISPR associated protein 9)-mediated genome editing

## Abstract

CRISPR–Cas technologies contribute to enhancing our understanding of plant gene functions, and to the precise breeding of crop traits. Here, we review the latest progress in plant genome editing, focusing on emerging CRISPR–Cas systems, DNA-free delivery methods, and advanced editing approaches. By illustrating CRISPR–Cas applications for improving crop performance and food quality, we highlight the potential of genome-edited crops to contribute to sustainable agriculture and food security.

## Highlights

• CRISPR–Cas technology facilitates plant functional genomics and crop breeding
• Emerging CRISPR–Cas systems provide opportunities for advanced genomic manipulations
• DNA-free editing approaches enable the obtention of transgene-free crops
• Edited crops with improved performance and product quality are available in the market
• Genome-edited crops provide solutions for sustainable agriculture and food security

## 1 Introduction

Centuries of directed evolution have fixed large parts of crop genomes, which constrains the breeding of crops with enhanced performance to secure global food supply and address ever-changing consumer needs. CRISPR–Cas technology offers a means to introduce new genetic variations into elite cultivars and create desirable traits absent in wild relatives (Li et al., 2024a). Precise and efficient gene editing is obtained using a customizable guide RNA (gRNA) that directs Cas nucleases or their engineered derivatives to specific genomic regions. Notably, the Cas9 system from *Streptococcus pyogenes* is widely used, but newer systems like Cas12 and OMEGA offer promising versatility. Advanced CRISPR–Cas tools and new methods for their component delivery now allow to obtain transgene-free crops with improved traits. Proof-of-concept studies have demonstrated enhancements in water and nutrient efficiency, stress tolerance, yield, and nutritional profiles in various crops. CRISPR–Cas technology also facilitates the domestication of orphan crops, which are crucial for local food security. By thoroughly discussing recent CRISPR–Cas technology advances for crop bioengineering, this review provides a roadmap for achieving more sustainable agriculture and tailor-made foods.

## 2 CRISPR–cas systems for genome editing

### 2.1 Cas9 systems

**FIGURE 1:**
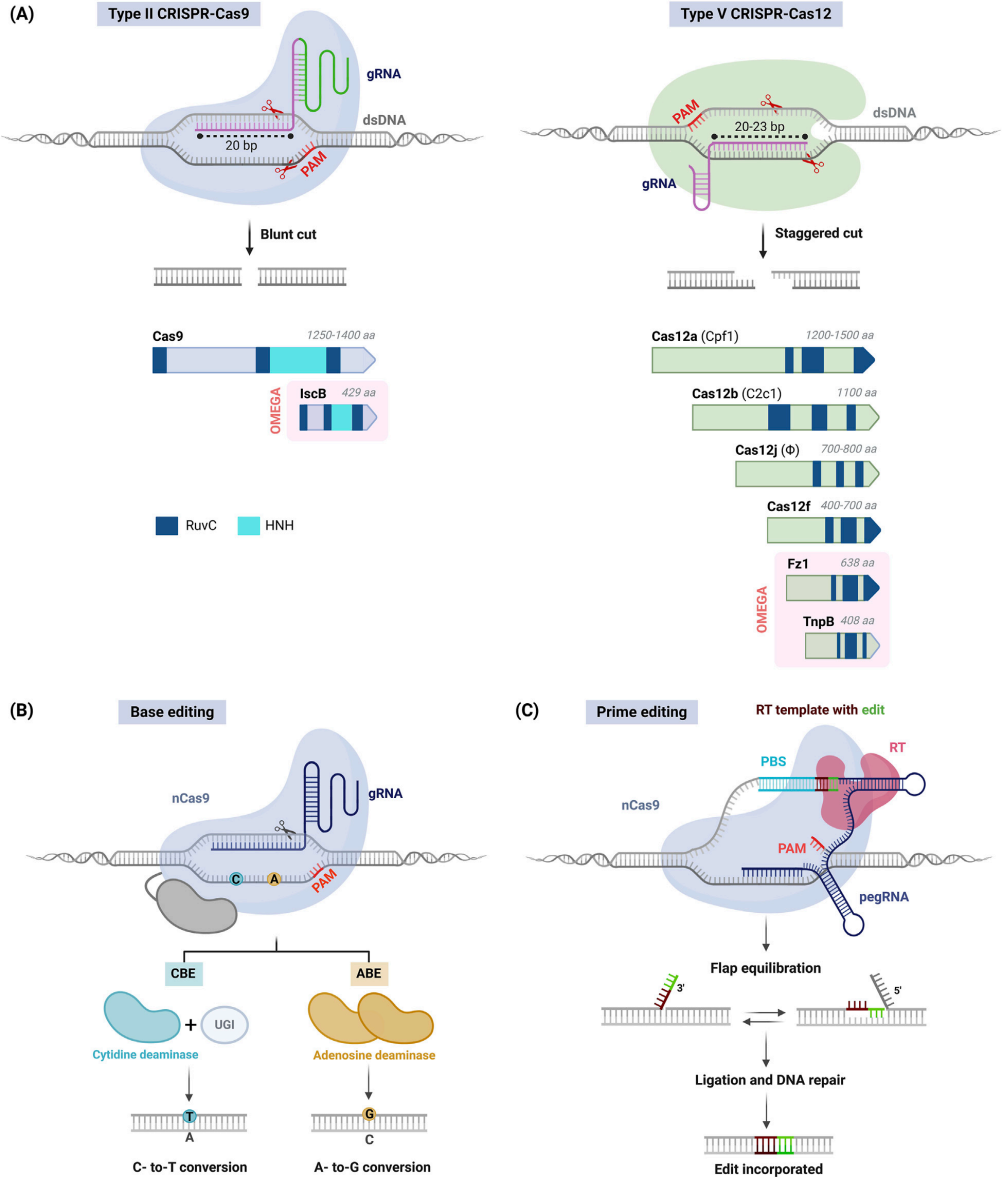
CRISPR–Cas systems for the precise engineering of plant genomes. **(A)** Gene editing using CRISPR–Cas systems. Upon guide RNA (gRNA) binding and recognition of a target site nearby a protospacer adjacent motif (PAM), Cas9 (left) or Cas12 (right) nucleases catalyze blunt or staggered DNA cuts, respectively, that lead to random indel production (top). The diversity of Cas9 and Cas12 families (bottom); nucleases are represented approximately to scale, including domain architecture and protein size (aa, amino acids); RuvC-like and HNH nuclease domains are shown. OMEGA family nucleases are highlighted in red background; Fz1, Fanzor 1; IscB, Insertion sequences Cas9-like B; TnpB, Transposon-associated protein B. **(B)** Base editing. A Cas9 nickase (nCas9) is fused to a cytidine (left) or adenosine (right) deaminase to obtain a cytosine base editor (CBE) or adenosine base editor (ABE), respectively; UGI, uracil DNA glycosylase inhibitor. **(C)** Prime editing. A prime editing complex consists of nCas9 fused to a reverse transcriptase (RT) and an extended gRNA (pegRNA) that includes a 5′ protospacer, an RT template and a 3′ primer-binding site (PBS). nCas9 generates a nick in the non-target strand, and the PBS functions as an RT template. Uncoupling of the prime editor leaves a 3′ flap (edited PAM strand) and a 5′ flap (original strand), and DNA repair mechanisms incorporate the desired edits into the genome.

The CRISPR–Cas system of *S. pyogenes*, which includes the Cas9 effector (SpCas9), is the best known among systems that have been repurposed for genome editing (Type II, Figure 1A). This system uses an engineered gRNA where the 20-nucleotide variable region at its 5′ end can be customized to guide the recognition of target DNA sequences. The Cas9 protein binds to a conserved region within the gRNA, and target DNA specificity is ensured by a nearby protospacer adjacent motif (PAM), specifically 5′-NGG-3′ for SpCas9. Upon binding, Cas9 induces a blunt-end double-strand break (DSB) that triggers cellular DNA repair mechanisms (Que et al., 2019). In plants, non-homologous end joining (NHEJ) is the primary repair pathway, often causing small insertions or deletions (indels) that can efficiently disrupt genes for knock-out purposes. Alternatively, homology-directed repair (HDR) allows the introduction of specific sequence changes using a homologous DNA template but is less efficient and more challenging in plants.

The SpCas9 effectiveness is limited by the need for a suitable G-rich PAM in the target gene, the potential for unintended changes in the genome (i.e., off-targets), and the large size of the nuclease (≈4.2 kb), which may constrain efficient delivery into plant cells. The development of Cas9 variants with diverse PAM requirements and reduced off-target effects has largely addressed these issues (Kulcsár et al., 2017). Additionally, Cas9 orthologs from bacteria and archaea have been identified by exploring the natural diversity of CRISPR–Cas systems (Koonin et al., 2023), though their complex PAM requirements often limit their use in plants.

### 2.2 Cas12 systems

The first significant alternative to Cas9 was Cas12a (formerly Cpf1) (Type V, Figure 1A). Cas12a recognizes a T-rich PAM (5′-TTTV-3′) that uses short gRNAs for sequence targeting, largely simplifying gRNA design and delivery. Moreover, its capacity to autonomously process gRNA precursors enables efficient multiplex editing. Unlike Cas9, Cas12a produces staggered DNA cuts that may enhance HDR-mediated knock-in. Several orthologs from the bacteria *Acidaminococcus* sp. BV3L6 (AsCas12a), *Francisella novicida* (FnCas12a) and *Lachnospiraceae bacterium* (LbCas12a) have been proven effective in plants, being LbCas12a particularly noted for its high editing activity (Malzahn et al., 2019).

The Cas12 family includes a variety of effectors with unique forms and functionalities (Figure 1A). Cas12b (formerly C2c1) is a variant that is smaller than Cas12a and produces long staggered cuts, thus showing promise in plant editing (Ming et al., 2020). Hypercompact CRISPR–Cas systems like Cas12j (CasΦ) and Cas12f are also being explored to overcome cargo constraints in expression vectors and facilitate delivery into plant cells (Li et al., 2023; Sukegawa et al., 2023).

### 2.3 Cas fusions: Base, prime, and exonuclease-assisted editors

HDR-mediated editing via conventional CRISPR–Cas systems is challenging due to difficulties for the *in vivo* delivery of exogenous DNA templates and the low rates of HDR in plant cells. As an alternative, base editing (BE) employs DNA deaminases fused to a Cas nickase (nCas) to induce specific base changes without producing DSBs (Li et al., 2024a). BE technology includes cytosine base editors (CBEs) that convert cytosine into uracil (resulting in C-to-T substitutions) and adenine base editors (ABEs) that transform adenine into inosine (resulting in A-to-G conversions) (Figure 1B). CBEs and ABEs can be combined in a dual system producing all four base transitions (C to T, A to G, T to C, and G to A) with high precision; additionally, use of optimized base editors resulted in efficient C-to-G and A-to-C editing in tomato and rice (Tian et al., 2022; Li et al., 2024c). Base editing has proven effective in plants, and its use with various Cas effectors has expanded the range of PAMs for gene targeting (Li et al., 2024a).

For more complex genome modifications, prime editing enables all 12 types of single-base changes (including four transitions and eight transversions), and small insertions or deletions (Vats et al., 2024). This approach involves an engineered nCas9-reverse transcriptase (RT) effector that is targeted to specific genomic loci by gRNA whose sequence is extended to include a primer-binding site (PBS) and an RT template with the desired edits (Figure 1C). Upon nicking the target site, single-stranded DNA hybridizes with the PBS and acts as a primer for the RT, which then transfers the desired edits from the gRNA to the non-target strand. Finally, the transcribed edits are integrated into the genome by cell DNA repair mechanisms and replication. Optimized prime editor and gRNA architectures have enabled the practical applications in plants, albeit limited to the introduction of short sequences (50-100 bases). Combined use of prime editors and serine integrases enables larger gene insertions up to 40 kilobases (Anzalone et al., 2022), though further work is needed for plant applications.

Another approach to producing large deletions involves fusing Cas effectors with exonucleases that generate long overhangs at the DSB to enhance HDR. Fusion of 5′-to-3′ exonucleases to Cas9 and Cas12a enables precise, scar-free insertion of several kilobases in plants, thus leading to stable and heritable gene knock-ins (Schreiber et al., 2024). This exonuclease-assisted editor system seems a promising tool for gene targeting purposes.

### 2.4 RNA-guided nucleases and recombinases from transposable elements

The Obligate Mobility Element-Guided Activity (OMEGA) system comprises a family of small, transposon-encoded RNA-guided nucleases considered the evolutionary ancestors of Cas9 and Cas12 effectors (Altae-Tran et al., 2021) (Figure 1A). The effector proteins are exceptionally diverse and exceptionally hypercompact compared to canonical CRISPR effectors. Insertion sequences Cas9-like B (IscB) is a tiny nuclease resembling Cas9 in structure and function that has been engineered for precise genome editing (Han et al., 2023). Transposon-associated protein B (TnpB) proteins are regarded as the precursors of Cas12 and has recently been adapted for effective editing of plant genomes (Li et al., 2024b). The newly discovered Fanzor (Fz) proteins, commonly found in eukaryotes and with remote homology to TnpB and Cas12, have also been adapted for genome editing (Saito et al., 2023), highlighting the potential of these novel systems for future applications.

Additionally, recent work shows the utility of RNA-guided recombinases from transposable elements for producing large-scale, programmable genomic rearrangements (Durrant et al., 2024; Hiraizumi et al., 2024). IS110 are a family of minimal transposable elements encoding a recombinase and a non-coding bridge RNA that contains two internal loops for target and donor DNA binding. These loops can be independently reprogrammed to trigger sequence-specific DNA insertions, deletions or inversions via genetic recombination; by facilitating large-scale genomic rearrangements, the IS110 bridge RNA recombination is a promising tool to upgrade the field of genome design.

## 3 CRISPR–Cas delivery into plant cells

### 3.1 Transgenic approaches

**FIGURE 2:**
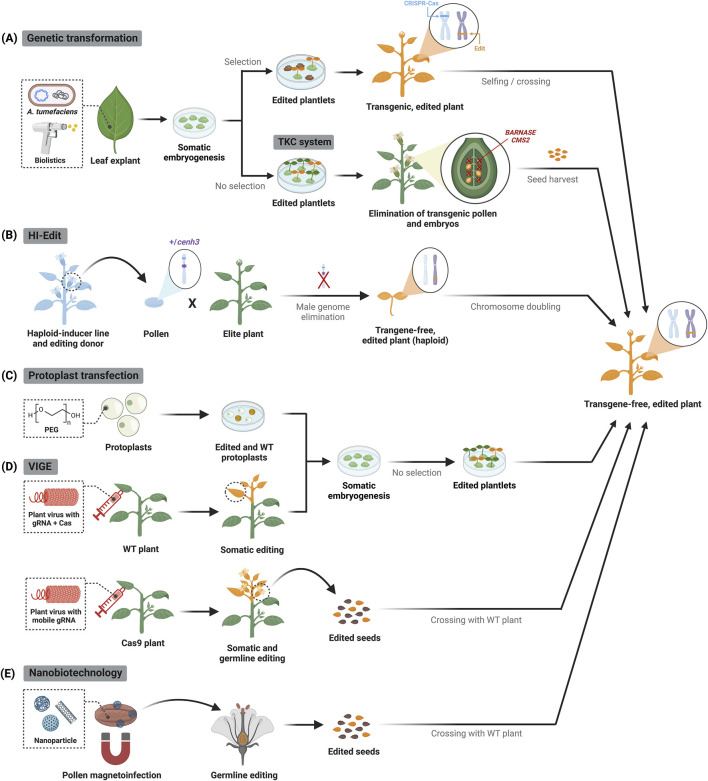
CRISPR–Cas delivery to plant cells and approaches to recover transgene-free edited plants. **(A)** Stable genetic transformation relies on tissue culture and marker-assisted selection to obtain genome-edited plants containing randomly integrated CRISPR–Cas transgene, which is then segregated by selfing or crossing. Alternatively, the transgene killer CRISPR (TKC) system triggers suicide gene expression (i.e., *BARNASE* and *CMS2*) during the reproductive stages, thus facilitating selection of transgene-free progeny. Edited plants or plant parts are highlighted in yellow. **(B)** Haploid-induced editing (HI-Edit) involves crossing haploid-inducer pollen containing CRISPR–Cas components with an untransformed elite plant, generating maternally edited, transgene-free haploid plants whose ploidy is restored by chemical treatment. **(C)** In protoplast transfection, CRISPR–Cas components (DNA plasmids or preassembled ribonucleoproteins) are transiently delivered into plant cells, which can be regenerated into edited plants. **(D)** In VIGE, viral vectors carrying either gRNAs and Cas nucleases, or mobile gRNAs are inoculated in wild-type (WT) or Cas9-expressing plants, respectively. Systemic spread of the viral infection generates somatic mutations; mobile gRNAs also induce heritable germline modifications bypassing tissue culture. The Cas9 transgene in the edited progeny is segregated out by crossing with WT plants. **(E)** Magnetic nanoparticles can deliver DNA constructs for CRISPR–Cas expression into pollen grains, which are used to pollinate WT plants and thus generate inheritable modifications.

Targeted genome editing requires delivering Cas proteins and gRNA into plant cells. Genetic transformation methods based on *Agrobacterium* spp. are routinely used for the stable integration of cassettes for CRISPR–Cas component expression in plant cells, which efficiently induce targeted genomic modifications (Liu et al., 2019) (Figure 2A, top panel). This procedure has been successfully applied in diverse plant species, although regenerating transformed cells into whole plants can sometimes be challenging and raises the risks for unwanted genomic changes like somaclonal variations. Alternatively, plants are transformed using biolistics by shooting particles coated with DNA plasmids for CRISPR–Cas expression into cells (Ozyigit and Yucebilgili Kurtoglu, 2020) (Figure 2A, top panel). This procedure is mainly applied in monocots, but the editing efficiency varies significantly between bombardments owing to inconsistent cargo loading.

*Agrobacterium*-mediated stable transformation approaches are exceptionally efficient and allow for the simultaneous modification of multiple genes. However, continuous expression of the editing components potentiates off-target events (Sturme et al., 2022), and the presence of an integrated CRISPR–Cas transgene may constrain the market release of edited crops. Integrated transgenes can be segregated out through selfing or crossing followed by identification of transgene-free offspring through PCR-mediated genotyping or visual selection using fluorescent markers or pigment reporters (Liu et al., 2019). Alternatively, the transgene killer CRISPR (TKC) system includes conditionally lethal genes in the T-DNA expression cassette to prevent the propagation of transgenic offspring, ensuring all surviving plants are transgene-free by the T_1_ generation (He et al., 2018) (Figure 2A, bottom panel). Haploid induction has also been applied to prevent the integration of editing components in the final product, with promising results in cereal crops. In the haploid-induced editing (HI-Edit) strategy, haploid-inducer pollen containing CRISPR–Cas components is crossed with a non-transgenic elite crop variety to yield transgene-free and edited maternal haploid offspring. The progeny is later subjected to chromosome doubling to obtain homozygous, doubled haploid plants with fixed genome traits (Kelliher et al., 2019) (Figure 2B).

### 3.2 Protoplast transfection

Genome integration of CRISPR–Cas components can be prevented by transient delivery into protoplasts, which are plant cells devoid of cell wall (Figure 2C). Besides DNA plasmids encoding CRISPR–Cas expression cassettes, it is possible to deliver preassembled Cas9-gRNA ribonucleoproteins that are immediately functional and get rapidly degraded, thus minimizing off-target effects (Zhang et al., 2021; Lin et al., 2022). While protoplast transfection is valuable for testing CRISPR set-ups and creating bi-allelic mutants from individual editing events, regenerating whole plants from protoplasts requires the development of species-specific protocols and is still a major bottleneck for its application in crop improvement.

### 3.3 Virus-induced genome editing (VIGE)

Plant viruses engineered for the transient delivery of CRISPR–Cas components into plant cells offer a flexible and cost-effective method for gene editing (Pasin et al., 2024). Viral vectors are locally introduced into plants and the systemic spread of the infection leads to highly efficient (≥70%) and rapid (3-7 days) gene editing (Uranga and Daròs, 2023) (Figure 2D). Because RNA viruses lack DNA replication intermediates, they are particularly suitable for generating edited plants without integration of viral genetic material into the plant genome. The current VIGE toolbox includes a variety of RNA viruses that have been successfully applied to express gRNAs for targeted editing in crops, though they commonly rely on transgenic plants that constitutively express the Cas9 nuclease (Uranga, 2024). This limitation can be addressed by negative-stranded RNA viruses that have large cargo capacities, which enables the simultaneous delivery of gRNAs and Cas nucleases into plant cells (Gao et al., 2019; Ma et al., 2020; Liu et al., 2023) (Figure 2D, top panel). Because most RNA viruses cannot access germline cells, strategies based on endogenous mobile RNAs are now being used to enhance heritable genome editing (Ellison et al., 2020) (Figure 2D, bottom panel).

### 3.4 Nanobiotechnology

Progress in nanobiotechnology has led to the development of diverse nanomaterials with low cell toxicity that can deliver biomolecules into plant cells for several purposes, including genetic engineering (Sanzari et al., 2019; Squire et al., 2023). Nanoparticle-mediated delivery of CRISPR–Cas components holds the promise to address current limitations of genome editing in plants by inducing targeted modifications in a species-independent manner (Demirer et al., 2021) (Figure 2E). However, further developments are needed to enhance loading of large CRISPR–Cas components into nanocarriers and their efficient delivery into plant cells by overcoming physical barriers, such as cell wall and membrane, or their internalization to the unwanted cell compartment, i.e., vacuolar sequestration.

## 4 Advancing gene editing approaches for crops

### 4.1 Multiplexing and high-throughput CRISPR–Cas editing

Multiplex genome editing allows multiple trait engineering by expression of Cas nucleases or their versions optimized for prime or base editing in combination with several gRNAs that target different genes (Cheng et al., 2023; Gupta et al., 2024) (Table 1). Targeting multiple genes in the same metabolic pathway has yielded significant agronomic improvements, such as isoflavone-enrichment in soybean (Zhang et al., 2020b), and increased lycopene content in tomato (Li et al., 2018b). Multiplex genome editing is also useful in improving agronomic traits under multigenic control. For instance, in tomato, simultaneous editing of *PHYTOENE SYNTHASE 1* (*PSY1*), the R2R3-MYB transcription factor *MYB12*, and *STAYGREEN 1* (*SGR1*) produced diverse fruit colors (Yang et al., 2022).

**TABLE 1:** Relevant applications of CRISPR–Cas editing in crop improvement.

Trait	Crop	Target gene(s)^a^	Result	References
Product quality	Rice	*Waxy*	Amylose-free waxy starch	Zeng et al. (2020)
	Wheat	α-, ω- and γ-gliadin genes	Reduced gluten content	Sánchez-León et al. (2024)
	Soybean	*FAD2-1A, FAD2-1B*	Increased oleic acid	Do et al. (2019)
		*F3H1, F3H2, FNSII*	Increased isoflavone content	Zhang et al. (2020b)
	Tomato	*Blc, LCY-B1, LCY-B2, LCY-E*	Increased lycopene content	Li et al. (2018b)
		*PSY1, SGR1, MYB12*	Recolored fruits	Yang et al. (2022), Uranga et al. (2024)
		*7-DR2*	Increased provitamin D_3_ content	Li et al. (2022a)
	Potato	*GBSS1*	Amylose-free waxy starch	Andersson et al. (2017)
	Canola	*A06.GTR2*	Reduced glucosinolate content	He et al. (2022)
		*FAD2*	Increased oleic acid	Huang et al. (2020)
	Strawberry	*ZIPs1.1*	Increased sugar content	Xing et al. (2020)
Plant architecture and yield	Rice	*Hd1*	Early flowering	Zhou et al. (2024)
	Wheat	*ARF12*	Increased grain size and weight, spike architecture	Kong et al. (2023)
	Maize	*GA20ox3*	Reduced height	Zhang et al. (2020a)
		*CLE7, FCP1*	Increased ear size and grain number	Liu et al. (2021)
	Tomato	*CLV3, S, SP*	Increased fruit size, branching and compact growth	Rodríguez-Leal et al. (2017)
		*SP, SP5G, ER*	Early flowering, compact growth	Kwon et al. (2020)
	Cotton	*TFL1*	Early flowering, compact growth	Wang et al. (2024)
	Apple and pear	*TFL1*	Early flowering	Charrier et al. (2019)
Stress resistance	Rice	*TFIIA*	Resistance to bacterial blight	Gupta et al. (2023)
	Wheat	*EDR1, MLO*	Resistance to powdery mildew	Zhang et al. (2017), Li et al. (2022b)
		*eIF4E*	Broad-spectrum resistance to viruses	Kan et al. (2023)
	Cucumber	*eIF4E*	Broad-spectrum resistance to viruses	Chandrasekaran et al. (2016)
	Melon	*eIF4E*	Broad-spectrum resistance to viruses	Pechar et al. (2022)
	Tomato	*TOM1*	Tobamovirus resistance	Ishikawa et al. (2022)
	Sweet orange	*LOB1*	Resistance to citrus canker	Su et al. (2023)

^a^ 7-DR, 7-dehydrocholesterol reductase; A06. GTR2, Glucosinolate transporter A06. GTR2; ARF12, Auxin responsive factor 12; Blc, Beta-lycopene cyclase; CLE7, Clavata 3/Esr-related 7; CLV3, Clavata 3; EDR1, Enhanced disease resistance 1; eIF4E, Eukaryotic translation initiation factor 4E; ER, Erecta; F3H, Flavanone 3-hydroxylase; FAD2, Fatty acid desaturase 2; FCP1, Fucoxanthin-chlorophyll binding protein 1; FNSII, Flavone synthase II; GA20ox3, Gibberellin 20-oxidase 3; GBSS1, Granule-bound starch synthase 1; Hd1, Heading date 1; LCY-B, Lycopene beta cyclase; LCY-E, Lycopene epsilon cyclase; LOB1, Lateral organ boundaries 1; MLO, Mildew resistance locus O; MYB12, R2R3-MYB, Transcription factor 12; PSY1, Phytoene synthase 1; S, Compound inflorescence; SGR1, Staygreen 1; SP, Self-pruning; SP5G, Self-pruning 5G; TFIIA, Transcription initiation factor IIA; TFL1, Terminal flower 1; TOM1, Tobamovirus multiplication 1; ZIPs1.1, Zinc transporter protein s1.1.

Multiplex genome editing is particularly useful for targeting homologous genes in polyploid species (Table 1). In the tetraploid potato, targeting *GRANULE-BOUND STARCH SYNTHASE 1* (*GBSS1*) in protoplasts followed by regeneration led to new lines with altered starch content (Andersson et al., 2017). In the hexaploid wheat, Cas9-mediated loss-of-function mutations of *MILDEW RESISTANCE LOCUS O* (*MLO*) homeologues resulted in elite wheat varieties with powdery mildew resistance and no growth penalties (Li et al., 2022b).

Another application of multiplex genome editing is the development of genome-wide mutant libraries. In tomato, gRNA libraries targeting 72 genes enabled the identification of 30 edited genes in two mutant populations (Jacobs et al., 2017), and a library targeting 990 transcription factors was used to develop 487 mutant lines (Bi et al., 2023). In maize, targeting 743 genes associated with agronomic and nutritional traits facilitated their functional annotation (Liu et al., 2020a). In rice, a genome-wide library of 25,604 gRNAs allowed to target 12,802 genes, resulting in a collection of more than 14,000 mutant lines, with 33% homozygous and 10% biallelic edits (Meng et al., 2017). Additionally, in canola, a gRNA library targeting 10,480 genes generated more than 1,100 edited plants that all together harbored modifications in 52.2% of the targets (He et al., 2023).

### 4.2 Directed evolution

Directed evolution relies on *de novo* generation of novel variants of a gene-of-interest using methods including CRISPR–Cas-based editing, to rapidly obtain improved agricultural traits under conditions of selective pressure. In rice, selected gRNAs have been employed to induce mutations in spliceosome-related proteins and generate plants with impaired splicing showing altered salt stress responses (Butt et al., 2021). Base editing systems have also been successfully applied in rice for directed evolution purposes. For instance, targeting *ACETYL COA CARBOXILASE* (*OsACCase*) using base editors with libraries of 63 or 141 gRNAs generated herbicide-resistant rice plants (Kuang et al., 2020; Liu et al., 2020b). Using an equivalent approach, saturation mutagenesis of *ACETOLACTATE SYNTHASE 1* (*OsALS1)* yielded mutants with varying levels of herbicide tolerance, which was eventually introduced into a commercial rice cultivar (Li et al., 2020). Furthermore, newly developed approaches using orthogonal base editors and multiple gRNAs enabled high *OsACCase* diversification and identification of evolved alleles conferring strong herbicide resistance (Zhang et al., 2023a).

### 4.3 CRISPR–Cas editing of non-coding regions

Editing of coding sequences may result in null alleles that compromise plant development or viability. Conversely, modifying cis-regulatory regions of specific genes can lead to alleles with varying expression levels and dosage-dependent phenotypes (Table 1). This approach was first demonstrated by targeting gene promoters of tomato, which led to novel mutant lines showing a continuum range of fruit size, inflorescence branching, and plant architecture phenotypes (Rodríguez-Leal et al., 2017). In maize, editing the promoter region of *CLAVATA 3/ESR-RELATED 7* (*CLE7*), which controls kernel number, resulted in a range of different ear morphologies (Liu et al., 2021). In rice, multiplex editing of flowering gene promoters achieved up to 88% mutation efficiency, providing new phenotypic variation in the timing of flowering induction (Zhou et al., 2024). Similar strategies have been employed to modify traits like amylose content in rice, fruit size in tomato, and sweetness in strawberry (Xing et al., 2020; Zeng et al., 2020; Wang et al., 2021). All these promoter-editing strategies rely on Cas9, which creates small indels that may be insufficient to alter the functionality of cis-regulatory regions. Alternatively, Cas12a and exonuclease-assisted editors may be more suitable for editing promoters and other non-coding sequences, given their ability to produce large deletions, as recently proven by applications to modify grain starch content in rice (Zhou et al., 2023), or the functional inactivation of a microRNA gene in tomato (Liu et al., 2024).

### 4.4 *De novo* domestication

Wild and underutilized relatives of crops can be highly adapted to harsh or changing environments. However, breeding efforts are required to integrate them into current food production systems by improving agronomic traits already selected in mainstream crops. Many domestication traits are monogenic and well-characterized (Zhang et al., 2023b), making them ideal candidates for CRISPR–Cas-based editing to rapidly improve wild relatives or underutilized crops by *de novo* domestication strategies. Pioneering studies in *Solanum pimpinellifolium* reported that the inactivation of genes associated with tomato domestication generated lines with increased fruit number and size, high vitamin C and lycopene levels, while maintaining disease resistance and salt tolerance (Li et al., 2018a; Zsögön et al., 2018). Similarly, groundcherry (*Physalis pruinosa*) lines with improved fruit size and number, and architecture were obtained by editing of *SELF-PRUNING* (*SP*), *SELF-PRUNING 5G* (*SP5G*), *CLAVATA 1* (*CLV1*), and *ERECTA* (*ER*) (Lemmon et al., 2018; Kwon et al., 2020). By leveraging knowledge of domestication genetics of cultivate rice varieties, targeted editing of key homologues of the wild relative *Oryza alta* resulted in lines with enhanced agronomic traits (Yu et al., 2021). Notably, a point mutation in the *IDEAL PLANT ARCHITECTURE 1* (*IPA1*) gene resulted in plants with improved yield traits. Additionally, a multiplex editing approach to target the four homeologs of *GRAIN NUMBER, PLANT HEIGHT AND HEADING DATE 7* (*GHD7*) resulted in varieties with enhanced flowering habits.

## 5 Applications for crop trait breeding

### 5.1 Improving plant architecture and yield

Targeting genes related to plant architecture and crop yield can transform the productivity and resource management of future agricultural practices (Table 1). In tomato, CRISPR–Cas applications have generated new phenotypes beneficial for cultivation and harvesting. For instance, stacking *SP, SP5G* and *ER* mutant alleles resulted in rapid-flowering plants with highly compact architecture suited for urban farming (Kwon et al., 2020). Editing of *TERMINAL FLOWER 1* (*TFL1*) produced a variety of architectures in cotton (Wang et al., 2024), and led to early flowering in apple and pear (Charrier et al., 2019), a strategy that might be useful to reduce juvenility periods in fruit trees. In maize, loss-of-function alleles of *GIBBERELLIN 20-OXIDASE 3* (*GA20ox3*) resulted in semi-dwarf plants with high grain yield and improved drought tolerance (Zhang et al., 2020a). In wheat, targeting genes related to grain size, weight, and spike architecture have increased biomass production by up to 11.1% (Kong et al., 2023).

### 5.2 Improving product quality

Enhancing product quality to meet consumer demands involves eliminating allergens or toxic compounds, as well as enriching the nutritional profile for improved food functionality (Tuncel et al., 2023) (Table 1). Wheat gluten may trigger immunogenic disorders in humans; wheat lines with edited α-, ω- and γ-gliadin genes showed a 97.7% reduction in gluten content, which could be used in gluten-free diets (Sánchez-León et al., 2024). In canola, new *A06. GTR2* alleles decreased seed content of glucosinolate while maintaining its beneficial role in plant defense (He et al., 2022). Editing of *FATTY ACID DESATURASE 2* (*FAD2*) in *Camelina sativa*, soybean, and canola increased oleic acid content and reduced polyunsaturated fatty acids in seeds (Jiang et al., 2017; Do et al., 2019; Huang et al., 2020). In melon, editing the NAC transcription factor non-ripening (*NAC-NOR*) delayed ripening and extended postharvest life without affecting nutritional quality (Liu et al., 2022). Fruit color is an important qualitative trait for fresh market tomatoes; recolored yellow, pink, and brown tomatoes were generated by inactivating genes involved in pigment accumulation in fruits (Yang et al., 2022; Uranga et al., 2024). Additionally, knocking out a tomato specific isoform of *7-DEHYDROCHOLESTEROL REDUCTASE 2* (*7-DR2*) increased provitamin D_3_, providing biofortified tomatoes as a way to tackle vitamin D deficiency (Li et al., 2022a).

After commercial release in 2019 of edited soybean (Calyno™, Calyxt Inc) with high oleic oil obtained by the transcription activator-like effector nucleases (TALENs) technology, a number of edited crops whose product quality was enhanced by CRISPR–Cas approaches are being sold on the market too (Table 2). Edited tomatoes with high amounts of γ-aminobutyric acid (GABA), a health-promoting compound, reached the Japanese market in 2021 as Sicilian Rouge^®^ (Waltz, 2022). Among others, in lettuce, editing of polyphenol oxidase (PPO) genes to prevent enzymatic discoloration resulted in the release of the commercial variety GVR-108XL with extended shelf life (GreenVenus LLC, 2022).

**TABLE 2:** CRISPR-edited crops available in the market.

Product	Target gene^a^	Improved feature^b^	Company	Market status^c^	References
Tomato Sicilian Rouge^®^	*GAD3*	High GABA	Sanatech Seed	Released, 2021 (Japan)	Waltz (2022)
Lettuce GVR-108XL	*PPO*	Non-browning	GreenVenus LLC	Released, 2023 (U.S.)	GreenVenus LLC (2022)
Maize Waxy^®^	*GBSS1*	Amylose-free waxy starch	Corteva Agriscience	Released, 2023 (U.S.)	Gao et al. (2020)
Pennycress CoverCress^TM^	*FAE1*, *TT8*	Low erucic acid, low fiber	CoverCress Inc	FDA-approved, pre-commercial	Hartnell et al. (2022)
Mustard greens Conscious Greens^®^	*Myrosinase*	Reduced pungency	Pairwise	Released, 2023 (U.S.)	Karlson et al. (2022)
Banana	*PPO*	Non-browning	Tropic Biosciences	Released, 2023 (Phillipines)	Tripathi et al. (2024)

^a^ *FAE1, Fatty acid elongation 1; GAD3, Glutamate decarboxylase 3; GBSS1, Granule-bound starch synthase 1; PPO, Polyphenol oxidase; TT8, Transparent testa 8*.

^b^ GABA, γ-aminobutyric acid.

^c^ FDA, U.S., food and drug administration.

### 5.3 Enhancing disease resistance

Gene editing offers a rapid and promising solution for enhancing disease resistance in crops (Table 1). In wheat, resistance to powdery mildew was achieved by generating knock-out alleles of *ENHANCED DISEASE RESISTANCE 1* (*EDR1*) genes (Zhang et al., 2017) and by creating novel *MLO* alleles (Li et al., 2022b). In rice, prime editing of the *TRANSCRIPTION INITIATION FACTOR IIA* (*TFIIA*) transcription factor provided broad-spectrum resistance against bacterial blight (Gupta et al., 2023). Similarly, broad-spectrum resistance to viruses was achieved in cucumber, melon, and wheat by editing the key susceptibility gene *EUKARYOTIC TRANSLATION INITIATION FACTOR 4E* (*eIF4E*) (Chandrasekaran et al., 2016; Pechar et al., 2022; Kan et al., 2023). Additionally, quadruple knock-out of *TOBAMOVIRUS MULTIPLICATION 1* (*TOM1*) homologues in tomato provided broad resistance to tobamoviruses, including the emerging pathogen tomato brown rugose fruit virus (ToBRFV) (Ishikawa et al., 2022). In sweet orange, editing of the *LATERAL ORGAN BOUNDARIES 1* (*LOB1*) gene generated resistance to citrus canker (Su et al., 2023). These advancements in crop protection are crucial for ensuring stable yields and securing food availability.

## 6 Outlooks

Collectively classified as New Genomic Techniques (NGTs), CRISPR–Cas-based editing approaches have already driven the development and market release of genome-edited foods (Table 2). Nonetheless, regulatory policies vary globally (Tuncel et al., 2023; Regnault-Roger, 2024). For instance, edited varieties with small genomic changes and no insertion of foreign DNA are regarded as conventionally bred plants in the USA, the UK, Japan and Argentina. Conversely, in Europe, NGT-derived plants are currently classified as genetically-modified organisms under the Directive 2001/18/EC of the European Parliament, however a revision of this strict regulatory framework is ongoing.

The continuous advancements in the CRISPR toolbox offer unique opportunities for genetic crop improvement. Beyond gene knock-out and small sequence editing, modification of non-coding elements as well as epigenome-mediated gene regulation are now possible. However, continued research is necessary to enable transient reagent delivery and avoid issues related to stable transformation or tissue culture. Additionally, making DNA-free genome editing more efficient and widely applicable in precision breeding could speed up the generation of transgene-free crops with improved performance and more sustainable production.
